# A Clinical Evaluation Denture Adhesives Used by Patients With Xerostomia: Erratum

**DOI:** 10.1097/01.md.0000475642.70710.bc

**Published:** 2015-12-28

In the article “A Clinical Evaluation Denture Adhesives Used by Patients With Xerostomia”,^1^ which appeared in Volume 94, Issue 7 of *Medicine*, statistically significant different values in Table 4 were presented incorrectly. The article has since been corrected online.
